# A widespread internal resonance phenomenon in functionally graded material plates with longitudinal speed

**DOI:** 10.1038/s41598-018-37921-9

**Published:** 2019-02-13

**Authors:** Y. F. Zhang, J. T. Liu

**Affiliations:** College of Aerospace Engineering, Shenyang Aerospace University, Shenyang, 110136 China

## Abstract

A widespread internal resonance phenomenon is detected in axially moving functionally graded material (FGM) rectangular plates. The geometrical nonlinearity is taken into account with the consideration of von Kármán nonlinear geometric equations. Using d’Alembert’s principle, governing equation of the transverse motion is derived. The obtained equation is further discretized to ordinary differential equations using the Galerkin technique. The harmonic balance method is adopted to solve the above equations. Additionally, stability analysis of steady-state solutions is presented. Research shows that a one-to-one internal resonance phenomenon widely exists in a large range of constituent volume distribution in moving FGM plates. Moreover, it is found that this internal resonance phenomenon can easily happen even though the FGM plates are under extremely small external excitation or with very large damping.

## Introduction

In order to meet the demanding requirements for comprehensive behavior of engineering structures in modern industries, a group of Japanese materials researchers composed a new type of non-uniform composite materials in the mid-1980s, namely, functional Gradient Materials (FGMs)^1^. The advantage of FGMs is that physical properties have no mutation in the materials. In recent years, FGM structures have been widely applied in defense industry, ships, aerospace and other high-tech fields. Therefore, the mechanical behavior analysis of FGM structures has attracted increasing attention.

In practical applications, FGM plates are usually important structural element. Dynamics investigation of FGM plates plays significant role in structure design. However, dynamics analyses of FGM plates are still not large^2–4^. Among them, some studies were carried out on *linear* dynamics of FGM plates^5–7^. On the other hand, literature on *nonlinear* dynamics of FGM plates is very limited. Wang and coauthors analyzed imperfection and piezoelectricity effect on non-linear behavior of FGM plates^8,9^. Hao *et al*.^10^ presented nonlinear vibration study of FGM plates; quasi periodic, periodic and chaotic vibrations were mainly discussed. Nonlinear vibrations of embedded FGM plates was discussed by Duc *et al*.^11^ based on the Runge-Kutta method. Based on the Lagrange method, Alijani *et al*.^12^ studied nonlinear dynamics of FGM plates using pseudo-arc-length continuation technique. Wang and Zu^13^ presented broadband vibration of traveling piezoelectric FGM plates. Zhang *et al*.^14^ discussed chaotic vibration of shear deformable FGM plates employing the technique of multiple scales. Adopting single-mode approximation, Allahverdizadeh *et al*.^15^ analyzed the existence condition of periodic solutions to FGM plates. FGM plates with cracks were considered by Yang *et al*.^16^, who discussed nonlinear frequencies and transient response of the structure. Recently, using the classical plate theory, Wang and Zu^17^ considered non-linear steady-state response of moving FGM plates in fluid.

Structural internal resonance is a unique nonlinear phenomenon in engineering system. If any of the natural frequencies of structures are commensurable, internal resonance can occur. In this condition, mode interaction becomes strong, and hence, significant. The system energy is continuously converted between the two coupled modes, and the amplitude and phase change periodically. Thus, understanding the mechanism of internal resonance in structural elements such as beams, plates and shells is of importance for the design and application of these structures. Some studies have focused on the internal resonance phenomenon in these structures, for example, internal resonance in metal plates^18^, beams^19^, cylindrical shells^20^, fluid-conveying pipes^21^, and strips and strings^22^.

Longitudinally moving continuums have attracted much attention in the past twenty years owing to their broad spectrum of use in various engineering fields such as robotic manipulators, extrusion processes, and so on. One-dimensional longitudinally moving continuums including strings, belts and beams have been widely investigated^23–30^. Two-dimensional longitudinally moving continuums such as plates were also studied^31,32^. Hatami *et al*.^33^ studied natural frequencies of longitudinally moving viscoelastic plates by utilizing the method of finite strip. Wang and Zu^34^ carried out analytical study on free vibration of traveling plates immerged in liquid. Banichuk *et al*.^35^ studied the stability of traveling plates with a constant speed; their concentration was focused on transverse vibration of the plates. Marynowski^36^ carried out linear dynamics study on longitudinally traveling viscoelastic plates of Levy type. Wang *et al*.^37–39^ studied deeply dynamic characteristics of moving plates with fluid-structure interaction.

Literature review indicates that research on dynamics of longitudinally moving plates was mainly focused on *metal* plates. This paper studies dynamic characteristics of longitudinally moving FGM plates, and attention is particularly focused on the internal resonance behavior. On the base of d’Alembert’s principle, mathematical model of the system is developed by taking into account geometrical nonlinearity of von Kármán type. The obtained model is further discretized to ordinary differential equations using the Galerkin technique. The harmonic balance method is adopted to solve these equations. This study retains all original physical quantities in the parametric study, thus allowing intuitive understanding of physical parameter effects on the internal resonance behavior.

## Mathematical Modeling

**Figure 1:**
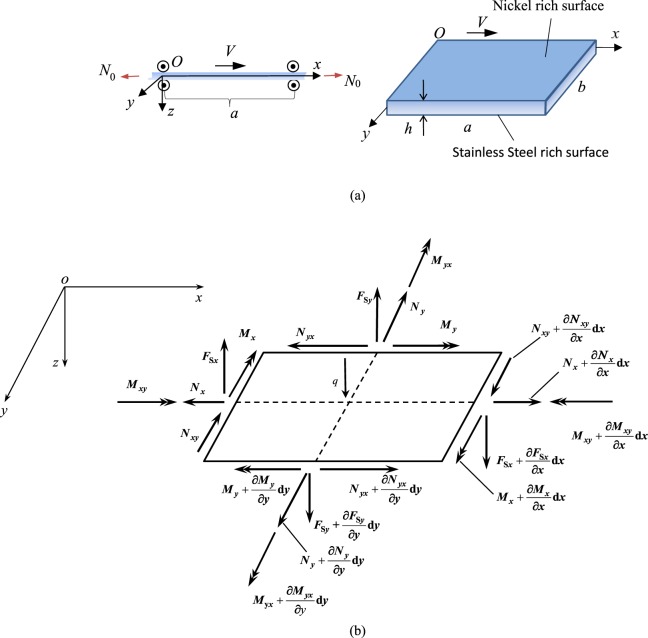
Schematic of moving FGM plate: (**a**) geometry and coordinate system; (**b**) Forces and moments acting on a plate element.

Consider a thin rectangular FGM plate made of stainless steel and nickel, which is simply supported at all edges and axially travels with a constant velocity *V*, as seen in Fig. 1a. The plate has the thickness *h*, width *b* and length *a*. A coordinate system is established with the origin *O* locating at the corner of the plate. Let *u*, *v* and *w* represent displacements of the plate mid-plane along *x-*, *y-* and *z-* axes from static equilibrium (*u* = *v* = *w* = 0), respectively. The forces and moments acting on a plate element are presented in Fig. 1b. For simplification, the infinitesimal plate element is represented by its middle surface. Additionally, a tension per unit width along the *x-*axis, denoted by *N*_0_, is loaded on the plate.

For a FGM plate, its effective material properties are written as^40,41^:

1 $$P(z)={P}_{Ni}\,{V}_{Ni}(z)+{P}_{S}\,{V}_{S}(z)$$

in which *P*_*S*_ and *P*_*Ni*_ are material properties of stainless steel and nickel, respectively; *V*_*S*_ and *V*_*Ni*_ denote volume fractions of stainless steel and nickel, respectively.

The relation between both volume fractions should be

2 $${V}_{Ni}+{V}_{S}=1$$

The constituent volume fraction is considered to vary smoothly along the *z*-axis and satisfy power law distribution. For nickel, it is given by

3 $${V}_{S}(z)={(\frac{z}{h}+\frac{1}{2})}^{N}$$

where *N* ∈ [0, ∞) denote the power-law exponent.

Therefore, the general mass density *ρ*(*z*), Young’s modulus *E*(*z*) and Poisson’s ratio *μ*(*z*) of the FGM plate are

4 $$\mu (z)=({\mu }_{S}-{\mu }_{Ni})\,{(\frac{z}{h}+\frac{1}{2})}^{N}+{\mu }_{Ni}$$

5 $$E(z)=({E}_{S}-{E}_{Ni})\,{(\frac{z}{h}+\frac{1}{2})}^{N}+{E}_{Ni}$$

6 $$\rho (z)=({\rho }_{S}-{\rho }_{Ni})\,{(\frac{z}{h}+\frac{1}{2})}^{N}+{\rho }_{Ni}$$

According to the classical thin plate theory, we have^42^

7 $${\varepsilon }_{x}={\varepsilon }_{x}^{0}+z{\chi }_{x},\,{\varepsilon }_{y}={\varepsilon }_{y}^{0}+z{\chi }_{y},\,{\gamma }_{xy}={\gamma }_{xy}^{0}+2z{\chi }_{xy}$$

where *ε*_*x*_, *ε*_*y*_ and ${\gamma }_{xy}$ denote strains of an arbitrary point, *χ*_*xy*_, *χ*_*y*_ and *χ*_*x*_ the torsion and curvature changes of middle plane, ${\varepsilon }_{x}^{0}$, ${\varepsilon }_{y}^{0}$ and ${\gamma }_{xy}^{0}$ the mid-plane strains, *z* the distance of an arbitrary point to the mid-plane.

Geometric relations of the von Kármán nonlinear theory are^43,44^

8 $$\{{\varepsilon }_{x}^{0},\,{\varepsilon }_{y}^{0},\,{\gamma }_{xy}^{0}\}=\{\frac{\partial u}{\partial x}+\frac{1}{2}{(\frac{\partial w}{\partial x})}^{2},\,\frac{\partial v}{\partial y}+\frac{1}{2}{(\frac{\partial w}{\partial y})}^{2},\,\frac{\partial v}{\partial x}+\frac{\partial u}{\partial y}+\frac{\partial w}{\partial x}\frac{\partial w}{\partial y}\}$$

9 $$\{{\chi }_{x},\,{\chi }_{y},\,{\chi }_{xy}\}=\{-\frac{{\partial }^{2}w}{\partial {x}^{2}},\,-\frac{{\partial }^{2}w}{\partial {y}^{2}},\,-\frac{{\partial }^{2}w}{\partial x\partial y}\}$$

For a FGM plate, stress-strain relationships are given by

10 $${\boldsymbol{\sigma }}={\bf{Q}}\cdot {\boldsymbol{\varepsilon }}$$

where

11 $${{\boldsymbol{\sigma }}}^{{\rm{T}}}=[{\sigma }_{x}\,{\sigma }_{y}\,{\tau }_{xy}],\,{\bf{Q}}=[\begin{array}{ccc}{Q}_{{\rm{11}}} & {Q}_{{\rm{12}}} & {\rm{0}}\\ {Q}_{{\rm{21}}} & {Q}_{{\rm{22}}} & {\rm{0}}\\ {\rm{0}} & {\rm{0}} & {Q}_{{\rm{66}}}\end{array}],\,{{\boldsymbol{\varepsilon }}}^{{\rm{T}}}{\boldsymbol{=}}[{\varepsilon }_{x}\,{\varepsilon }_{y}\,{\gamma }_{xy}]$$

in which *τ*_*xy*_ stands for in-plane shear stress, *σ*_*x*_ and *σ*_*y*_ the normal stresses, *Q*_*ij*_ (*i*, *j* = 1, 2, 6) the reduced stiffness components.

Reduced stiffnesses are expressed as:

12 $${Q}_{{\rm{11}}}={Q}_{{\rm{22}}}=\frac{E(z)}{[1-\mu {(z)}^{2}]}$$

13 $${Q}_{{\rm{12}}}={Q}_{{\rm{21}}}=\frac{E(z)\cdot \mu (z)}{[{\rm{1}}-\mu {(z)}^{2}]}$$

14 $${Q}_{{\rm{66}}}=\frac{E(z)}{{\rm{2}}[{\rm{1}}+\mu (z)]}$$

The resultant forces and moments of the FGM plate take the form of^42,45^

15 $$[\begin{array}{c}{N}_{x}\\ {N}_{y}\\ {N}_{xy}\end{array}]={\int }_{-h/2}^{h/2}[\begin{array}{c}{\sigma }_{x}\\ {\sigma }_{y}\\ {\tau }_{xy}\end{array}]\,{\rm{d}}z$$

16 $$[\begin{array}{c}{M}_{x}\\ {M}_{y}\\ {M}_{xy}\end{array}]={\int }_{-h/2}^{h/2}[\begin{array}{c}{\sigma }_{x}\\ {\sigma }_{y}\\ {\tau }_{xy}\end{array}]\,z{\rm{d}}z$$

Substituting Eqs (7) and (10) in Eqs (15) and (16) leads to the constitutive relations

17 $${\boldsymbol{{\rm N}}}={\bf{S}}\cdot {\boldsymbol{\varepsilon }}$$

with

18 $${{\boldsymbol{{\rm N}}}}^{{\rm{T}}}=[\begin{array}{cccccc}{N}_{x} & {N}_{y} & {N}_{xy} & {M}_{x} & {M}_{y} & {M}_{xy}\end{array}]$$

19 $${{\boldsymbol{\varepsilon }}}^{{\rm{T}}}=[\begin{array}{cccccc}{\varepsilon }_{x}^{{\rm{0}}} & {\varepsilon }_{y}^{{\rm{0}}} & {\gamma }_{xy}^{0} & {\chi }_{x} & {\chi }_{y} & {\chi }_{xy}\end{array}]$$

and

20 $${\bf{S}}=[\begin{array}{cccccc}{A}_{11} & {A}_{12} & 0 & {B}_{11} & {B}_{12} & 0\\ {A}_{{\rm{12}}} & {A}_{{\rm{22}}} & 0 & {B}_{{\rm{12}}} & {B}_{{\rm{22}}} & 0\\ 0 & 0 & {A}_{{\rm{66}}} & 0 & 0 & {B}_{{\rm{66}}}\\ {B}_{11} & {B}_{12} & 0 & {D}_{11} & {D}_{12} & 0\\ {B}_{{\rm{12}}} & {B}_{{\rm{22}}} & 0 & {D}_{{\rm{12}}} & {D}_{{\rm{22}}} & 0\\ 0 & 0 & {B}_{{\rm{66}}} & 0 & 0 & {D}_{{\rm{66}}}\end{array}]$$

in which *A*_*ij*_, *B*_*ij*_ and *D*_*ij*_ (*i*, *j* = 1, 2, 6) denote stiffness coefficients. Their expressions take the form

21 $${A}_{ij}={\int }_{-h/2}^{h/2}{Q}_{ij}{\rm{d}}z$$

22 $${B}_{ij}={\int }_{-h/2}^{h/2}{Q}_{ij}z{\rm{d}}z$$

23 $${D}_{ij}={\int }_{-h/2}^{h/2}{Q}_{ij}{z}^{2}{\rm{d}}z$$

On the base of the d’Alembert principle, we can derive the dynamic equilibrium equation governing the transverse vibration of a moving FGM plate:

24 $$\begin{array}{c}{\int }_{-\,\frac{h}{2}}^{\frac{h}{2}}\rho \frac{{{\rm{d}}}^{2}w}{{\rm{d}}{t}^{2}}{\rm{d}}z-\frac{{\partial }^{2}{M}_{x}}{\partial {x}^{2}}-2\frac{{\partial }^{2}{M}_{xy}}{\partial x\partial y}-\frac{{\partial }^{2}{M}_{y}}{\partial {y}^{2}}-({N}_{x}+{N}_{0})\frac{{\partial }^{2}w}{\partial {x}^{2}}\\ \,-\,{N}_{y}\frac{{\partial }^{2}w}{\partial {y}^{2}}-2{N}_{xy}\frac{{\partial }^{2}w}{\partial x\partial y}+c(\frac{\partial w}{\partial t}+V\frac{\partial w}{\partial x})+F(x,y,t)=0\end{array}$$

in which *c* stands for damping coefficient.

The derivative of the first term in Eq. (24) takes the form

25 $$\frac{{{\rm{d}}}^{2}w}{{\rm{d}}{t}^{2}}=\frac{{\partial }^{2}w}{\partial {t}^{2}}+2V\frac{{\partial }^{2}w}{\partial x\partial t}+{V}^{2}\frac{{\partial }^{2}w}{\partial {x}^{2}}$$

The transverse external excitation *F*(*x*, *y*, *t*) in Eq. (24) is harmonic point load

26 $$F(x,y,t)={F}_{0}\,\cos ({\omega }t)\delta (x-{x}_{0})\delta (y-{y}_{0})$$

in which *δ* and *F*_0_ denote Dirac delta function and force amplitude, respectively, *ω* the excitation frequency, *x*_0_ and *y*_0_ the in-plane coordinates. The load is applied at *x*_0_ = *a*/2 and *y*_0_ = *b*/2 in this study.

Employing Eqs (7), (10), (15 and 16), (25) and (26) in Eq. (24) gives the governing equation in term of *w*

27 $$\begin{array}{c}{D}_{11}\frac{{\partial }^{4}w}{\partial {x}^{4}}+(2{D}_{12}+4{D}_{66})\frac{{\partial }^{4}w}{\partial {x}^{2}\partial {y}^{2}}+{D}_{22}\frac{{\partial }^{4}w}{\partial {y}^{4}}-{N}_{0}\frac{{\partial }^{2}w}{\partial {x}^{2}}+c(\frac{\partial w}{\partial t}+V\frac{\partial w}{\partial x})\\ \,+\,{\int }_{-\frac{h}{2}}^{\frac{h}{2}}\rho (\frac{{\partial }^{2}w}{\partial {t}^{2}}+2V\frac{{\partial }^{2}w}{\partial x\partial t}+{V}^{2}\frac{{\partial }^{2}w}{\partial {x}^{2}}){\rm{d}}z-2{A}_{66}\frac{\partial w}{\partial x}\frac{\partial w}{\partial y}\frac{{\partial }^{2}w}{\partial x\partial y}\\ \,+\,\frac{1}{2}(4{B}_{66}-4{B}_{12}){(\frac{{\partial }^{2}w}{\partial x\partial y})}^{2}+\frac{1}{2}(-2{B}_{12}-4{B}_{66})\frac{\partial w}{\partial x}\frac{{\partial }^{3}w}{\partial x\partial {y}^{2}}\\ \,+\,\frac{1}{2}(4{B}_{12}-4{B}_{66})\frac{{\partial }^{2}w}{\partial {x}^{2}}\frac{{\partial }^{2}w}{\partial {y}^{2}}+\frac{1}{2}(-2{B}_{12}-4{B}_{66})\frac{\partial w}{\partial y}\frac{{\partial }^{3}w}{\partial {x}^{2}\partial y}\\ \,+\,\frac{1}{2}[-{A}_{22}\frac{{\partial }^{2}w}{\partial {y}^{2}}{(\frac{\partial w}{\partial y})}^{2}-{A}_{12}\frac{{\partial }^{2}w}{\partial {x}^{2}}{(\frac{\partial w}{\partial y})}^{2}-{A}_{12}\frac{{\partial }^{2}w}{\partial {y}^{2}}{(\frac{\partial w}{\partial x})}^{2}\\ \,-\,{A}_{11}{(\frac{\partial w}{\partial x})}^{2}\frac{{\partial }^{2}w}{\partial {x}^{2}}-2{B}_{22}\frac{{\partial }^{3}w}{\partial {y}^{3}}\frac{\partial w}{\partial y}-2{B}_{11}\frac{\partial w}{\partial x}\frac{{\partial }^{3}w}{\partial {x}^{3}}]\\ \,+\,{F}_{0}\,\cos ({\omega }t)\delta (x-{x}_{0})\delta (y-{y}_{0})=0\end{array}$$

in which shear and normal stress components are neglected^46^.

## Approximate Analytical Solutions

In this study, we focus on the one-to-one internal resonance between the first two modes. Accordingly, the displacement that exactly satisfies the simply supported boundary condition is given by

28 $$w(x,y,t)={A}_{\bar{m},\bar{n}}(t)\,\sin (\frac{\bar{m}\pi x}{a})\,\sin (\frac{\bar{n}\pi y}{b})+{A}_{\bar{j},\bar{k}}(t)\,\sin (\frac{\bar{j}\pi x}{a})\,\sin (\frac{\bar{k}\pi y}{b})$$

where $\bar{m}$, $\bar{j}$, $\bar{n}$ and $\bar{k}$ denote the mode numbers; ${A}_{\bar{m},\bar{n}}(t)\,$ and ${A}_{\bar{j},\bar{k}}(t)\,$ stand for generalized coordinates with respect to time *t*.

Using the Galerkin method, the weight functions are given by

29 $${F}_{p}(x,y)=\{\begin{array}{ll}\sin \,(\bar{m}\pi x/a)\,\sin \,(\bar{n}\pi y/b) & p=1\\ \sin \,(\bar{j}\pi x/a)\,\sin \,(\bar{k}\pi y/b) & p=2\end{array}$$

The Galerkin procedure takes the form of

30 $$\langle Eq.\,(27),\,{F}_{p}\rangle ={\int }_{0}^{b}{\int }_{0}^{a}Eq.\,(27)\,{F}_{p}(x,y)\,{\rm{d}}x\,{\rm{d}}y$$

The derivation of Eq. (30) can be performed with the aid of *Mathematica* software^47^. Thus, we can derive the following nonlinear ordinary differential equations related to ${A}_{\bar{m},\bar{n}}(t)\,$ and ${A}_{\bar{j},\bar{k}}(t)\,$:

31 $$\{\begin{array}{c}{\ddot{A}}_{\bar{m},\bar{n}}(t)+{\bar{M}}_{1}{\dot{A}}_{\bar{m},\bar{n}}(t)+{\bar{M}}_{2}{\dot{A}}_{\bar{j},\bar{k}}(t)+{\bar{M}}_{3}{A}_{\bar{m},\bar{n}}(t)+{\bar{M}}_{4}{A}_{\bar{j},\bar{k}}(t)\\ \,+\,{\bar{M}}_{5}{A}_{\bar{m},\,\bar{n}}^{3}(t)+{\bar{M}}_{6}{A}_{\bar{m},\bar{n}}(t){A}_{\bar{j},\,\bar{k}}^{2}(t)+{\bar{M}}_{7}{A}_{\bar{m},\,\bar{n}}^{2}(t)+{\bar{M}}_{8}{A}_{\bar{j},\,\bar{k}}^{2}(t)+{\bar{M}}_{9}\,\cos \,(\omega t)=0\\ {\ddot{A}}_{\bar{j},\bar{k}}(t)+{\bar{S}}_{1}{\dot{A}}_{\bar{m},\bar{n}}(t)+{\bar{S}}_{2}{\dot{A}}_{\bar{j},\bar{k}}(t)+{\bar{S}}_{3}{A}_{\bar{m},\bar{n}}(t)\\ \,+\,{\bar{S}}_{4}{A}_{\bar{j},\bar{k}}(t)+{\bar{S}}_{5}{A}_{\bar{j},\,\bar{k}}^{3}(t)+{\bar{S}}_{6}{A}_{\bar{m},\,\bar{n}}^{2}(t){A}_{\bar{j},\bar{k}}(t)+{\bar{S}}_{7}{A}_{\bar{m},\bar{n}}(t){A}_{\bar{j},\bar{k}}(t)=0\end{array}$$

in which the over-dot stands for derivative to time; ${\bar{M}}_{i}$ and ${\bar{S}}_{j}$ (*i* = 1, 2, …, 9, *j* = 1, 2, …, 7) denote proper parameters which are presented in Appendix.

Introduce non-dimensional variables as follows

32 $$\tau ={\omega }_{\bar{m},\bar{n}}t,\,\Omega =\omega /{\omega }_{\bar{m},\bar{n}},\,{q}_{1}(\tau )={A}_{\bar{m},\bar{n}}(t)/h,\,{q}_{2}(\tau )={A}_{\bar{j},\bar{k}}(t)/h$$

where ${\omega }_{\bar{m},\bar{n}}$ is the fundamental natural frequency.

Employing Eq. (32) in Eq. (31) gives non-dimensional equations:

33 $$\{\begin{array}{c}{\ddot{q}}_{1}(\tau )={M}_{1}{\dot{q}}_{1}(\tau )+{M}_{2}{\dot{q}}_{2}(\tau )+{M}_{3}{q}_{1}(\tau )+{M}_{4}{q}_{2}(\tau )+{M}_{5}{q}_{1}^{3}(\tau )\\ \,\,\,\,+\,{M}_{6}{q}_{1}(\tau ){q}_{2}^{2}(\tau )+{M}_{7}{q}_{1}^{2}(\tau )+{M}_{8}{q}_{2}^{2}(\tau )+{M}_{9}\,\cos (\Omega \tau )\\ {\ddot{q}}_{2}(\tau )={S}_{1}{\dot{q}}_{1}(\tau )+{S}_{2}{\dot{q}}_{2}(\tau )+{S}_{3}{q}_{1}(\tau )+{S}_{4}{q}_{2}(\tau )\\ \,\,\,\,+\,{S}_{5}{q}_{2}^{3}(\tau )+{S}_{6}{q}_{1}^{2}(\tau ){q}_{2}(\tau )+{S}_{7}{q}_{1}(\tau ){q}_{2}(\tau )\end{array}$$

where *M*_*i*_ and *S*_*j*_ (*i* = 1, 2, …, 9, *j* = 1, 2, …, 7) denote proper coefficients deriving from the dimensionless transformation.

According to the harmonic balance method, the solutions of Eq. (33) can be expressed as

34 $${q}_{1}(\tau )={A}_{0}+\sum _{n=1}^{H}[{A}_{2n-1}\,\cos \,(n\Omega \tau )+{A}_{2n}\,\sin \,(n\Omega \tau )]$$

35 $${q}_{2}(\tau )={B}_{0}+\sum _{n=1}^{H}[{B}_{2n-1}\,\cos \,(n\Omega \tau )+{B}_{2n}\,\sin \,(n\Omega \tau )]$$

in which *A*_*n*_ and *B*_*n*_ (*n* = 0, 1, …, *H*) denote Fourier’s coefficients.

The time derivatives are expressed as

36 $${\dot{q}}_{1}(\tau )=\sum _{n=1}^{H}[\,-\,n\Omega {A}_{2n-1}\,\sin \,(n\Omega \tau )+n\Omega {A}_{2n}\,\cos \,(n\Omega \tau )]$$

37 $${\dot{q}}_{2}(\tau )=\sum _{n=1}^{H}[\,-\,n\Omega {B}_{2n-1}\,\sin \,(n\Omega \tau )+n\Omega {{{\rm B}}}_{2n}\,\cos \,(n\Omega \tau )]$$

38 $${\ddot{q}}_{1}(\tau )=\sum _{n=1}^{H}[\,-\,{n}^{2}{\Omega }^{2}{A}_{2n-1}\,\cos \,(n\Omega \tau )-{n}^{2}{\Omega }^{2}{A}_{2n}\,\sin \,(n\Omega \tau )]$$

39 $${\ddot{q}}_{2}(\tau )=\sum _{n=1}^{H}[\,-\,{n}^{2}{\Omega }^{2}{B}_{2n-1}\,\cos \,(n\Omega \tau )-{n}^{2}{\Omega }^{2}{B}_{2n}\,\sin \,(n\Omega \tau )]$$

Introducing Eqs (34–39) in Eq. (33) and extracting each harmonic terms, one can obtain 4*H* + 2 algebraic equations related to *A*_*n*_ and *B*_*n*_ (*n* = 0, 1, …, *H*). Setting *H* = 1, we have

40 $$\{\begin{array}{c}Fu{n}_{1}({A}_{0},{B}_{0},{A}_{1},{B}_{1},{A}_{2},{B}_{2},\Omega )=0\\ Fu{n}_{2}({A}_{0},{B}_{0},{A}_{1},{B}_{1},{A}_{2},{B}_{2},\Omega )=0\\ Fu{n}_{3}({A}_{0},{B}_{0},{A}_{1},{B}_{1},{A}_{2},{B}_{2},\Omega )=0\\ Fu{n}_{4}({A}_{0},{B}_{0},{A}_{1},{B}_{1},{A}_{2},{B}_{2},\Omega )=0\\ Fu{n}_{5}({A}_{0},{B}_{0},{A}_{1},{B}_{1},{A}_{2},{B}_{2},\Omega )=0\\ Fu{n}_{6}({A}_{0},{B}_{0},{A}_{1},{B}_{1},{A}_{2},{B}_{2},\Omega )=0\end{array}$$

in which *Fun*_*i*_ (*i* = 1, 2, …, 6) are expressions with respect to *A*_0_, *B*_0_, *A*_1_, *B*_1_, *A*_2_, *B*_2_ and *Ω*. Eq. (40) is very expatiatory and the forms of *Fun*_*i*_ are omitted here. From Eq. (40), one can obtain *A*_0_, *B*_0_, *A*_1_, *B*_1_, *A*_2_, *B*_2_ for a given *Ω*; substituting the results in Eqs (34) and (35) gives the solutions of *q*_1_ and *q*_2_.

## Stability of Steady State Analytical Solutions

For the purpose of analyzing the stability of steady state solutions, the following coordinate transformations are introduced:

41 $${q}_{1}(\tau )={A}_{0}+{\rm{\Delta }}{A}_{0}(\tau )+[{A}_{1}+{\rm{\Delta }}{A}_{1}(\tau )]\,\cos \,(\Omega \tau )+[{A}_{2}+{\rm{\Delta }}{A}_{2}(\tau )]\,\sin \,(\Omega \tau )$$

42 $${q}_{2}(\tau )={B}_{0}+{\rm{\Delta }}{B}_{0}(\tau )+[{B}_{1}+{\rm{\Delta }}{B}_{1}(\tau )]\,\cos (\Omega \tau )+[{B}_{2}+{\rm{\Delta }}{B}_{2}(\tau )]\,\sin (\Omega \tau )$$

where Δ*A*_*i*_(*τ*) and Δ*B*_*i*_(*τ*) (*i* = 0, 1, 2) mean perturbations.

Substituting Eqs (41 and 42) in Eq. (33), one may get a series of disturbance equations relating to Δ*A*_*i*_(*τ*) and Δ*B*_*i*_(*τ*) (*i* = 0, 1, 2). Their expressions are given by

43 $$\dot{{\bf{a}}}={\bf{f}}({\bf{a}},{\bf{s}},\tau )$$

where

44 $$\begin{array}{c}{\bf{a}}=\begin{array}{cccccc}[{\rm{\Delta }}{A}_{0}(\tau ) & {\rm{\Delta }}{\dot{A}}_{0}(\tau ) & {\rm{\Delta }}{B}_{0}(\tau ) & {\rm{\Delta }}{\dot{B}}_{0}(\tau ) & {\rm{\Delta }}{A}_{1}(\tau ) & {\rm{\Delta }}{\dot{A}}_{1}(\tau )\end{array}\\ \begin{array}{cccccccc} &  & {\rm{\Delta }}{B}_{1}(\tau ) & {\rm{\Delta }}{\dot{B}}_{1}(\tau ) & {\rm{\Delta }}{A}_{2}(\tau ) & {\rm{\Delta }}{\dot{A}}_{2}(\tau ) & {\rm{\Delta }}{B}_{2}(\tau ) & {\rm{\Delta }}{\dot{B}}_{2}(\tau )\end{array}{]}^{{\rm{T}}}\end{array}$$

45 $${\bf{s}}=\begin{array}{cccccc}[{A}_{0} & {B}_{0} & {A}_{1} & {B}_{1} & {A}_{2} & {B}_{2}\end{array}{]}^{{\rm{T}}}$$

46 $$\begin{array}{c}{\bf{f}}=\begin{array}{cccccc}[{f}_{1}({\bf{a}},{\bf{s}},\tau ) & {f}_{2}({\bf{a}},{\bf{s}},\tau ) & {f}_{3}({\bf{a}},{\bf{s}},\tau ) & {f}_{4}({\bf{a}},{\bf{s}},\tau ) & {f}_{5}({\bf{a}},{\bf{s}},\tau ) & {f}_{6}({\bf{a}},{\bf{s}},\tau )\end{array}\\ \begin{array}{cccccccc} &  & {f}_{7}({\bf{a}},{\bf{s}},\tau ) & {f}_{8}({\bf{a}},{\bf{s}},\tau ) & {f}_{9}({\bf{a}},{\bf{s}},\tau ) & {f}_{10}({\bf{a}},{\bf{s}},\tau ) & {f}_{11}({\bf{a}},{\bf{s}},\tau ) & {f}_{12}({\bf{a}},{\bf{s}},\tau )\end{array}{]}^{{\rm{T}}}\end{array}$$

At **a** = **0**, performing Taylor series expansion to **f** yields

47 $$\dot{{\bf{a}}}={\bf{Aa}}$$

in which **A** denote Jacobian matrix of the function **f** calculated in **a** = **0**.

For stable response, all real part of eigenvalues should be negative for the Jacobian matrix. Otherwise, the response is instable.

## Analytical and Numerical Results

To verify the present method, a comparison study is first made with the available reference for a simply supported stationary FGM plate made of Si_3_N_4_ and SUS304. The following parameters are used: *μ* = 0.28, *a* = 0.2 m, *b* = 0.2 m and *h* = 0.025 m. The frequency parameter ${\omega }^{\ast }=\omega {a}^{2}/h\sqrt{{\rho }_{m}(1-{\mu }^{2})/{E}_{m}}$ is calculated and compared with ref.^12^, as shown in Table 1. One can find perfect agreement between these results has been achieved.

**Table 1:** Comparison of frequency parameter ${\omega }^{\ast }=\omega {a}^{2}/h\sqrt{{\rho }_{m}(1-{\mu }^{2})/{E}_{m}}$ for stationary FGM plate at room temperature.

*N*	Present	Ref.^12^
Ceramic	13.175	13.173
0.5	9.111	9.068
1	7.985	7.948
2	7.205	7.140
Metal	5.699	5.698

In what follows, we deal with a nickel/stainless-steel FGM plate that travels in the *x*-axis direction. At room temperature, material parameters of stainless steel are obtained as *E*_*S*_ = 2.07788 × 10^11^ Nm^−2^, *μ*_*S*_ = 0.317756 and *ρ*_*S*_ = 8166 kg m^−3^, and those of nickel are obtained as *E*_*Ni*_ = 2.05098 × 10^11^ N m^−2^*, μ*_*Ni*_ = 0.31 and *ρ*_*Ni*_ = 8900 kg m^−3^. *a*, *b* and *h* of the FGM plate are 0.4 m, 0.1 m and 0.001 m, respectively. It is clear this is a thin plate due to *b*/*h* = 100.

**Figure 2:**
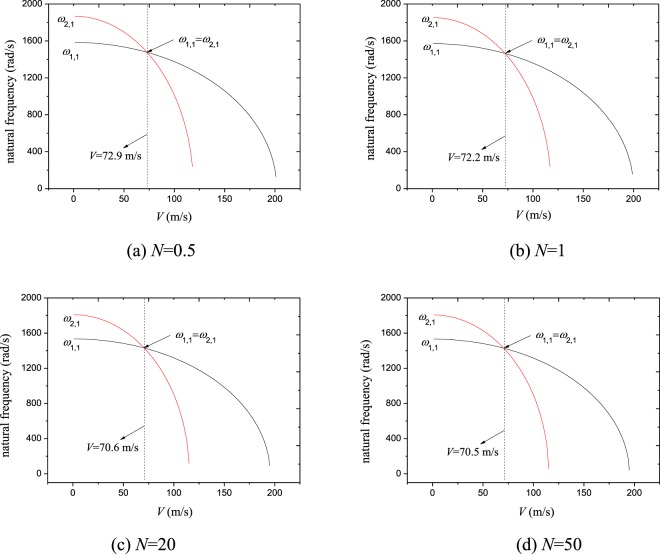
Variations of the first two natural frequencies against moving velocity for various power law exponents.

Figure 2 shows the change rule of the first two natural frequencies against moving velocity for a wide range of power-law exponents, i.e., *N* = 0.5 to *N* = 50. Here the pretension is set as *N*_0_ = 1000 N/m; natural frequencies *ω*_1,1_ and *ω*_2,1_ relate to the first mode ($\bar{m}=1$, $\bar{n}=1$) and second mode ($\bar{j}=2$, $\bar{k}=1$), respectively. From the figure, one may find that both *ω*_1,1_ and *ω*_2,1_ decrease with increasing moving speed of the FGM plate. However, their decrease rates are not always same. When the speed is small, i.e., *V* < 20 m/s, both decrease rates of the two frequencies have little difference. When *V* > 20 m/s, *ω*_2,1_ reduces quickly with the increase of moving speed; in contrast, *ω*_1,1_ still decreases slowly. This tendency results in the coincidence of these two natural frequencies at certain speeds, as seen in Fig. 2, and may result in 1:1 internal resonance. It is interesting to see that the coincidence of the lowest two natural frequencies appears under all of the considered power-law exponents. This demonstrates internal resonance exists in a broad range of constituent volume distribution in the moving FGM plate. In the present study, attention is mainly focused on this internal resonance behavior.

**Figure 3:**
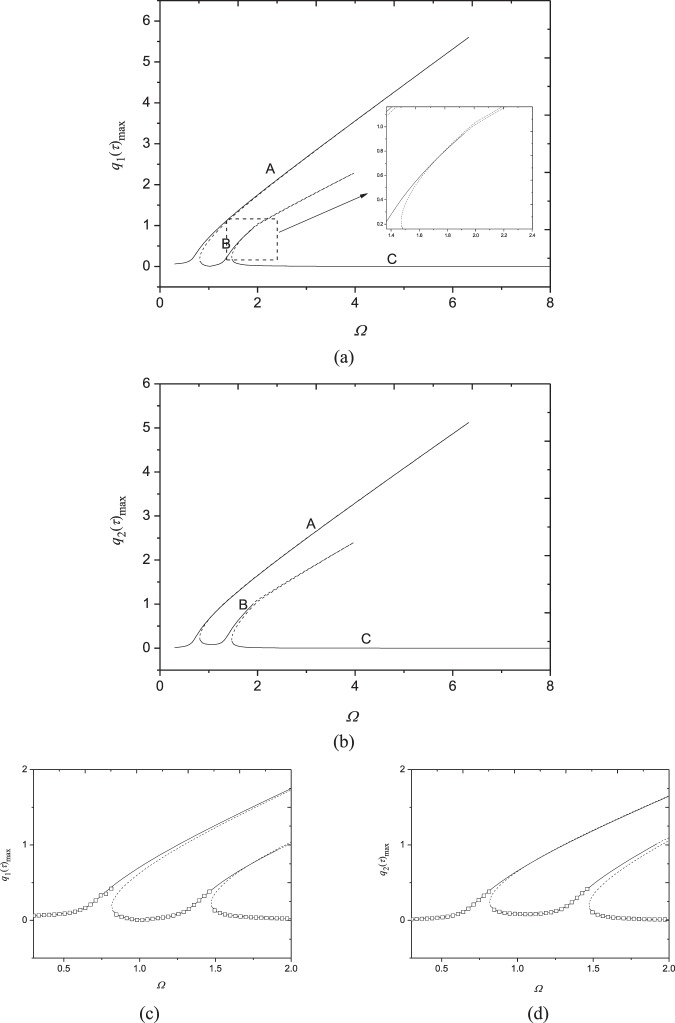
Frequency-response curves (*N*_0_ = 1000 N/m, *F*_0_ = 10N, *c* = 10 Ns/m^3^, *N* = 1, *V* = 72.2 m/s): (**a**) maximum of *q*_1_(*τ*); (**b**) maximum of *q*_2_(*τ*); (**c**) magnification of (**a**); (**d**) magnification of (**b**). □, numerical solution; —, stable response; ---, unstable response.

The frequency response relationships are investigated in Fig. 3 nearby the fundamental frequency. Here the parameters are *N*_0_ = 1000 N/m, *F*_0_ = 10 N, *c* = 10 Ns/m^3^, *V* = 72.2 m/s and *N* = 1. The fundamental natural frequency is obtained as *ω*_1,1_ = 1464.61 rad/s and the second natural frequency is *ω*_2,1_ = 1465.17 rad/s. It is seen that the ratio of them is *ω*_1,1_/*ω*_2,1_ ≈ 1, which may give rise to the 1:1 internal resonance.

From Fig. 3(a,b), one may find that each generalized coordinates have two peaks and they are different from zero nearby the fundamental natural frequency, which demonstrates the first two modes are excited simultaneously owing to the nonlinear coupling through one-to-one internal resonance. These frequency-response relationships haven’t been detected in moving homogenous plates before. Additionally, the frequency response curves exhibit hardening nonlinear characteristics. It is also seen that resonant amplitudes of each generalized coordinates are nearly same as each other due to 1:1 internal resonance. For the two peaks of each generalized coordinates, the first peak appears before the exact resonance condition *Ω* = 1 and lasts to *Ω* = 6.3290; the second one appears after *Ω* = 1 and its resonance region is narrower than the first one. Each mode has three stable branches (A, B and C). There are two saddle-node bifurcations on the peak A, i.e., at *Ω* = 6.3290 and *Ω* = 0.8272. The resonant response loses its stability at the first bifurcation point and then recovers stability at the second bifurcation point. After that, the second stable peak B appears; this stable branch lasts to *Ω* = 1.9154 and turns instable via Hopf bifurcation at this point. Coupled responses regain their stability at another bifurcation point at *Ω* =1.4741, resulting in the occurence of stable branch C. This branch relates to non-resonance response.

In order to verify the present analytical analysis, numerical solutions of Eq. (33) are solved by employing the Runge-Kutta method with assumed initial conditions ${q}_{1}(0)={q}_{2}(0)={\dot{q}}_{1}(0)={\dot{q}}_{2}(0)=0$. In Fig. 3(c,d), the analytical solutions are plotted together with numerical ones in a close-up view. It is seen that quite good agreement has been achieved between numerical and analytical solutions.

**Figure 4:**
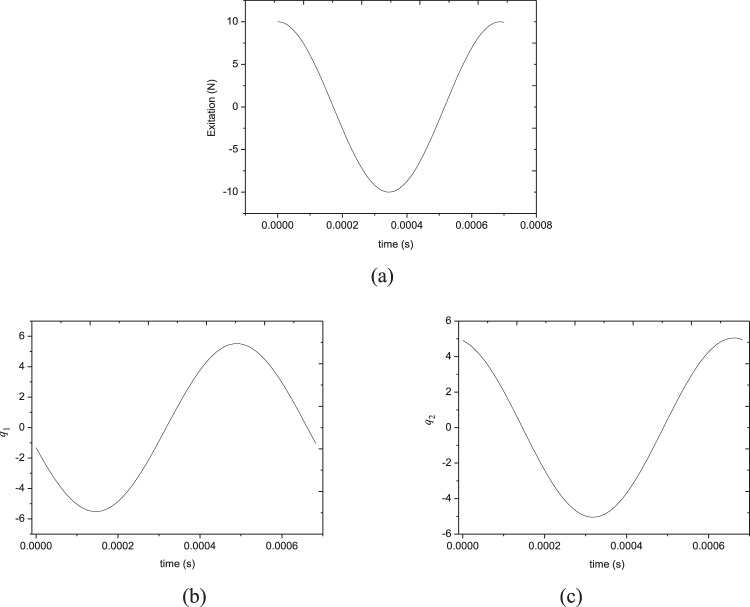
Time responses of generalized coordinates at *Ω* = 6.233 in Fig. 3: (**a**) external excitation; (**b**) generalized coordinate *q*_1_; (**c**) generalized coordinate *q*_2_.

**Figure 5:**
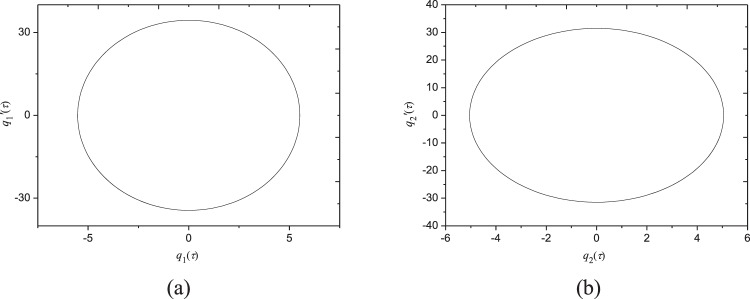
Phase plane diagram of generalized coordinates at *Ω* = 6.233 in Fig. 3.

Figures 4 and 5 gives the time responses and phase locus of *q*_1_ and *q*_2_, where the excitation frequency is *Ω* = 6.233. The excitation variation is shown in Fig. 4(a) for *F*_0_ = 10 N. These figures show that the system response is periodic, and the amplitudes of both sides of the plate are symmetrical during a vibration period. Particularly, the amplitudes of each generalized coordinates are nearly the same but the phase angle is π/2.

**Figure 6:**
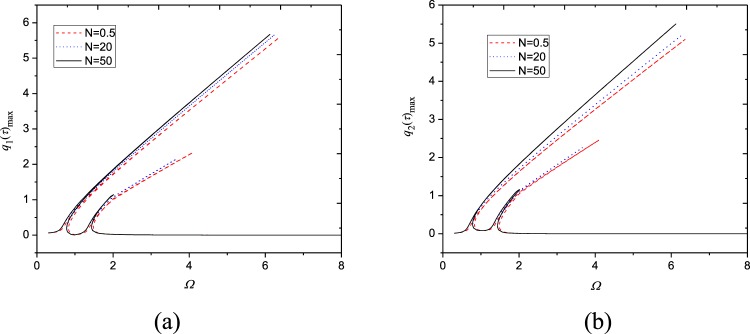
Effect of power-law exponent on frequency response of moving FGM plate (*N*_0_ = 1000 N/m, *c* = 10 Ns/m^3^, *V* = 72 m/s, *F*_0_ = 10 N): (**a**) maximum of *q*_1_(*τ*); (**b**) maximum of *q*_2_(*τ*).

Because power-law exponent is an important parameter which determines the configuration of FGM plates, its effect is particularly illuminated on vibration response of moving FGM plates in Fig. 6. As can be seen, power-law exponent has obvious influence on the resonance characteristics of FGM plates. When the power-law exponent rises, resonance amplitude of the plates increases accordingly. This is quite clear for the first peak in the frequency-response curves. Additionally, a trend is found that the second peak in the frequency-response curves shrinks with increasing power-law exponent.

**Figure 7:**
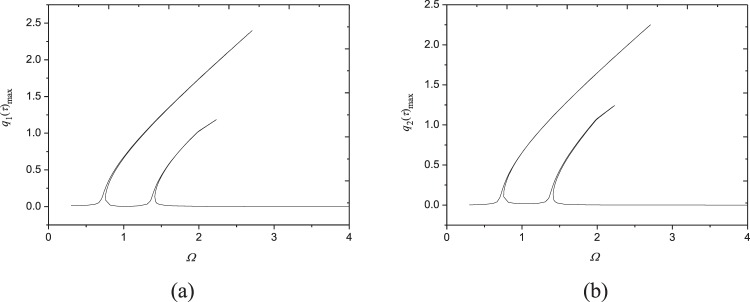
Frequency-response curves (*F*_0_ = 2N, *V* = 72.2 m/s, *N* = 1, *c* = 10 Ns/m^3^, *N*_0_ = 1000 N/m): (**a**) maximum of *q*_1_(*τ*); (**b**) maximum of *q*_2_(*τ*).

Figure 7 shows frequency-response curves under a small excitation *F*_0_ = 2N; the other parameters are kept the same as those in Fig. 3. As seen in Figs 3 and 7, both resonance amplitudes of the two modes decrease with the decreasing excitation amplitude. Also, as excitation amplitude decreases, hardening spring characteristics becomes weaker and weaker, and the resonance region gets narrower and narrower. It is worth noting that there still exist two obvious peaks on frequency response curves at very small excitation *F*_0_ = 2N. This shows the 1:1 internal resonance in the present system can be excited easily even under extremely small excitation, indicating the sensibility of moving FGM plates to external excitation.

**Figure 8:**
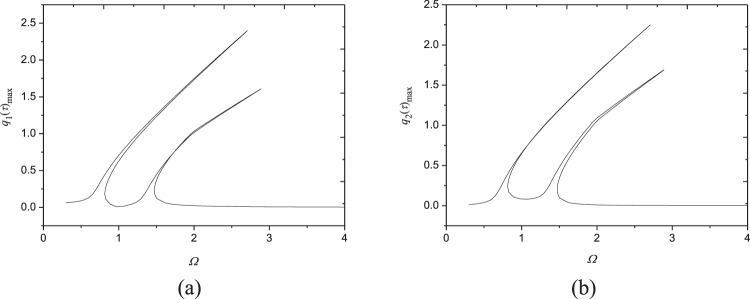
Frequency-response curves (*c* = 50 Ns/m^3^, *V* = 72.2 m/s, *F*_0_ = 10N, *N* = 1, *N*_0_ = 1000 N/m): (**a**) maximum of *q*_1_(*τ*); (**b**) maximum of *q*_2_(*τ*).

Increasing the damping coefficient from *c* = 10 Ns/m^3^ (Fig. 3) to *c* = 50 Ns/m^3^, Fig. 8 is generated. Comparing Figs 3 and 8 reveals that the resonance region of the system narrows with the increase of damping coefficient. Moreover, the larger damping coefficient leads to the smaller resonant amplitudes of each mode. It is also found the 1:1 internal resonance phenomenon can happen even though the damping is very large, by contrast, the internal resonance has gone in composite shells with large damping coefficient^48^.

**Figure 9:**
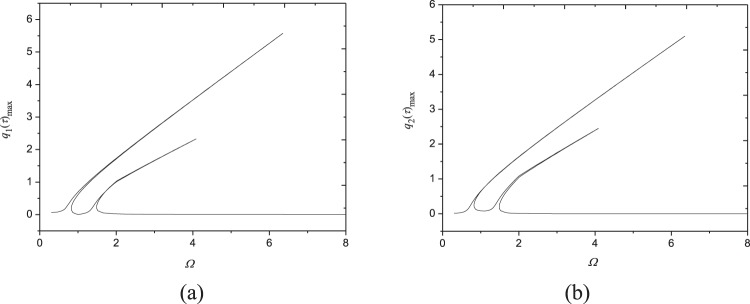
Frequency-response curves (*N* = 0.5, *N*_0_ = 10^3^ N/m, *c* = 10 Ns/m^3^, *V* = 72.85 m/s, *F*_0_ = 10N): (**a**) maximum of *q*_1_(*τ*); (**b**) maximum of *q*_2_(*τ*).

**Figure 10:**
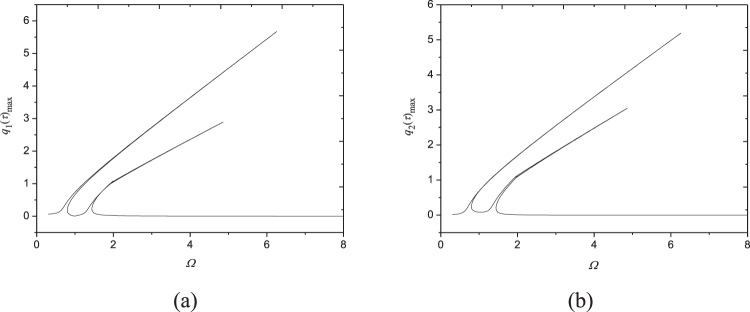
Frequency-response curves (*N* = 20, *N*_0_ = 10^3^ N/m, *c* = 10 Ns/m^3^, *V* = 70.61 m/s, *F*_0_ = 10N): (**a**) maximum of *q*_1_(*τ*); (**b**) maximum of *q*_2_(*τ*).

**Figure 11:**
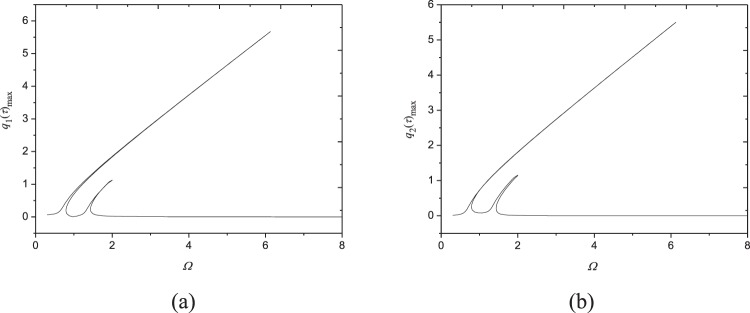
Frequency-response curves (*N* = 50, *N*_0_ = 10^3^ N/m, *c* = 10 Ns/m^3^, *V* = 70.47 m/s, *F*_0_ = 10N): (**a**) maximum of *q*_1_(*τ*); (**b**) maximum of *q*_2_(*τ*).

Figures 9–11 show frequency response relationships of FGM plates with various power law exponents in a wide range (from *N* = 0.5 to 50). All these figures are plotted in the conditions of *ω*_1,1_/*ω*_2,1_ = 1 for each power-law exponent to reveal the probable internal resonance phenomenon. The parameters used are shown under the corresponding figures. It is very interesting that 1:1 internal resonance appears in all these cases because each generalized coordinates generate extra peak. Comparing Figs 3, 9, 10 and 11 reveals that when the moving speed is within the range $V\in [70.5,\,72.9]$, 1:1 internal resonance can happen in a wide range of power-law exponent in FGM plates. Therefore, this nonlinear phenomenon needs to be considered when designing and applying moving FGM plates.

## Conclusions

A widespread one-to-one internal resonance phenomenon is detected in longitudinally moving FGM plates. On the base of d’Alembert’s principle, the equation of transverse vibration is derived with the consideration of von Kármán’s nonlinear geometrical relations. The approximately analytical analysis is conducted by using the Galerkin method together with the harmonic balance method. Results show that nonlinear frequency response relationship exhibits nonlinear hardening characteristics. The lowest two modes are excited simultaneously owing to the nonlinear coupling through one-to-one internal resonance. For moving FGM plates, the one-to-one internal resonance phenomenon may happen in a large range of constituent volume fraction. Furthermore, even extremely small excitation can excite this internal resonance.

## Supplementary information

Related Manuscript File
