# Supplementary material for: A widespread internal resonance phenomenon in functionally graded material plates with longitudinal speed
Source: Sci Rep. 2019 Feb 13;9:1907. doi: 10.1038/s41598-018-37921-9 (PMC6374398; doi:10.1038/s41598-018-37921-9)
Supplement: Supplementary file 1 — Related Manuscript File [file 41598_2018_37921_MOESM1_ESM.pdf]

A widespread internal resonance phenomenon in functionally graded material plates with longitudinal speed

Y. F. Zhang *, J. T. Liu

（*School of Aerospace Engineering, Shengyang Aerospace University, Shenyang* 110136, *China*）

Appendix

The coefficients in Eq. (31) are as follows for ($\bar{m}=1,\bar{n}=1$) and ($\bar{j}=2,\bar{k}=1$)

$$\bar{M}_1=\frac{c}{\int_{-\frac{h}{2}}^{\frac{h}{2}}\rho(z)\mathrm{dz}}$$

$$\bar{M}_2=-\frac{16V}{3a}$$

$$\bar{M}_3=\frac{\pi^4 D_{11}}{a^4\int_{-\frac{h}{2}}^{\frac{h}{2}}\rho(z)\mathrm{dz}}+\frac{2\pi^4 D_{12}}{a^2b^2\int_{-\frac{h}{2}}^{\frac{h}{2}}\rho(z)\mathrm{dz}}+\frac{4\pi^4 D_{66}}{a^2b^2\int_{-\frac{h}{2}}^{\frac{h}{2}}\rho(z)\mathrm{dz}}+\frac{\pi^2 N_0}{a^2\int_{-\frac{h}{2}}^{\frac{h}{2}}\rho(z)\mathrm{dz}}-\frac{\pi^2V^2}{a^2}+\frac{\pi^4 D_{22}}{b^4\int_{-\frac{h}{2}}^{\frac{h}{2}}\rho(z)\mathrm{dz}}$$

$$\bar{M}_4=-\frac{8cV}{3a\int_{-\frac{h}{2}}^{\frac{h}{2}}\rho(z)\mathrm{dz}}$$

$$\bar{M}_5=\left(\frac{3\pi^4A_{11}}{32a^4}+\frac{3\pi^4A_{12}}{16a^2b^2}-\frac{\pi^4A_{66}}{8a^2b^2}+\frac{3\pi^4A_{22}}{32b^4}\right)\Big/\int_{-\frac{h}{2}}^{\frac{h}{2}}\rho(z)\mathrm{dz}$$

$$\bar{M}_6=\left(\frac{3\pi^4A_{11}}{4a^4}+\frac{21\pi^4A_{12}}{16a^2b^2}-\frac{\pi^4A_{66}}{a^2b^2}+\frac{3\pi^4A_{22}}{16b^4}\right)\Big/\int_{-\frac{h}{2}}^{\frac{h}{2}}\rho(z)\mathrm{dz}$$

$$\bar{M}_7=\left(\frac{32\pi^2B_{11}}{9a^4}+\frac{160\pi^2B_{12}}{9a^2b^2}+\frac{32\pi^2B_{66}}{9a^2b^2}+\frac{32\pi^2B_{22}}{9b^4}\right)\Big/\int_{-\frac{h}{2}}^{\frac{h}{2}}\rho(z)\mathrm{dz}$$

$$\bar{M}_8=\left(\frac{3584\pi^2B_{11}}{45a^4}+\frac{512\pi^2B_{12}}{9a^2b^2}+\frac{1664\pi^2B_{66}}{45a^2b^2}+\frac{128\pi^2B_{22}}{45b^4}\right)\Big/\int_{-\frac{h}{2}}^{\frac{h}{2}}\rho(z)\mathrm{dz}$$

$$\bar{M}_9=\frac{4F_0}{ab\int_{-\frac{h}{2}}^{\frac{h}{2}}\rho(z)\mathrm{dz}}\sin(\frac{\pi x_0}{a})\sin(\frac{\pi y_0}{b}) \qquad \text{(A1)}$$

$$\bar{S}_1 = \frac{16V}{3a}$$

$$\bar{S}_2 = \frac{c}{\int_{-\frac{h}{2}}^{\frac{h}{2}} \rho(z)\mathrm{dz}}$$

$$\bar{S}_3 = \frac{8cV}{3a\int_{-\frac{h}{2}}^{\frac{h}{2}} \rho(z)\mathrm{dz}}$$

$$\bar{S}_4 = \frac{1}{a^4 b^4 \int_{-\frac{h}{2}}^{\frac{h}{2}} \rho(z)\mathrm{dz}} (\pi^4 a^4 D_{22} - 4\pi^2 a^2 b^4 V^2 \int_{-\frac{h}{2}}^{\frac{h}{2}} \rho(z)\mathrm{dz} + 4\pi^2 a^2 b^4 N_0 +$$

$$8\pi^4 a^2 b^2 D_{12} + 16\pi^4 a^2 b^2 D_{66} + 16\pi^4 b^4 D_{11})$$

$$\bar{S}_5 = \frac{\pi^4 (3a^4 A_{22} + 24a^2 A_{12} b^2 - 16a^2 A_{66} b^2 + 48A_{11} b^4)}{32a^4 b^4 \int_{-\frac{h}{2}}^{\frac{h}{2}} \rho(z)\mathrm{dz}}$$

$$\bar{S}_6 = \frac{\pi^4 (3a^4 A_{22} + 9a^2 A_{12} b^2 - 4a^2 A_{66} b^2 + 12A_{11} b^4)}{16a^4 b^4 \int_{-\frac{h}{2}}^{\frac{h}{2}} \rho(z)\mathrm{dz}}$$

$$\bar{S}_7 = \frac{128\pi^2 (2a^4 B_{22} + 25a^2 b^2 B_{12} - 4a^2 b^2 B_{66} + 5b^4 B_{11})}{45a^4 b^4 \int_{-\frac{h}{2}}^{\frac{h}{2}} \rho(z)\mathrm{dz}} \qquad (A2)$$
